# Colloidal silver ingestion and severe anemia – A case report

**DOI:** 10.1016/j.toxrep.2023.09.012

**Published:** 2023-09-20

**Authors:** Albin Stjernbrandt

**Affiliations:** Section of Sustainable Health, Department of Public Health and Clinical Medicine, Umeå University, 901 87 Umeå, Sweden

**Keywords:** Silver, Poisoning, Anemia, Case report

## Abstract

**Introduction:**

Colloidal silver ingestion as an alternative medicine treatment is becoming more common. This case report describes a patient with potential silver poisoning and severe anemia.

**Case description:**

A woman in her late sixties was transported to the emergency department because of progressive fatigue and nausea. She had been drinking 150 µg of colloidal silver daily for two to three weeks. Blood sampling revealed severe anemia (red blood cell count 48 g/L) and a whole-blood silver concentration of 20 µg/L. Liver function tests were abnormal and there were signs of incipient heart failure with increased pro-brain natriuretic peptide, troponin T, as well as pleural effusion. She was stabilized with blood transfusion and symptomatic treatment, to be discharged from the hospital after ten days. The patient improved over the following six weeks and the whole-blood silver concentration decreased to 3.3 µg/L after about three months.

**Conclusions:**

The case demonstrates the potential effects of silver intake on the hematopoietic, hepatic, and cardiovascular systems. This highlights the possible risks associated with emerging alternative medicine therapies.

## Highlights

• Silver is not an essential dietary metal, but oral exposure can occur through intake of fish and use of silver kitchen tools.
• Colloidal silver ingestion as an alternative medicine treatment is becoming more common.
• Our case report shows potential adverse effects of silver intake on the hematopoietic, hepatic, and cardiovascular systems.

## Graphical Abstract

**Figure:**
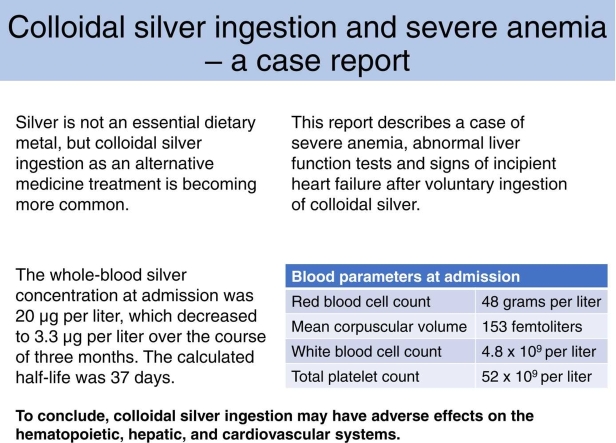

## 1 Introduction

Silver is a transition metal with high thermal and electrical conductivity that is mainly used in industrial applications but also production of jewelry, coins, and flatware. In the healthcare setting, silver is used as a local bacteriostatic agent in wound care. Silver is not an essential dietary metal, but oral exposure can occur through the intake of fish and seafood as well as the use of silver-containing dental fillings and kitchen tools. When ingested in higher doses, it can have a wide range of toxic effects on different internal organs, including the cardiovascular and hematopoietic systems as well as the liver, as shown mainly in animal studies [1], [2]. Silver can also be deposited in the skin, mucus membranes, and cornea and produce a blue-gray discoloration called argyria [3]. There have been reports of increasing use of silver compounds (i.e., colloidal silver) in alternative medicine [2], [3], [4] and this is cause for some concern since the effects have not been thoroughly studied in humans. This paper presents a case of potential silver poisoning by voluntary intake of a colloidal silver suspension.

## 2 Case description

A non-smoking and previously healthy woman in her late sixties presented to the emergency department at the local hospital due to progressive fatigue and nausea. She had been feeling increasingly weak during the last few days and her relatives had decided to call an ambulance when she was unable to walk without support. The patient had not experienced any bleeding from the nose, mouth, urinary tract, or bowel. At the presentation, she was lean and pale. There was no abnormal skin or corneal pigmentation. Bedside pulmonary auscultation revealed general crackling and reduced breath sounds over the lower right lobe. Other physical examination was unremarkable. Venous blood sampling revealed a low red blood cell (RBC) count of 48 g/L with an increased mean corpuscular volume as well as mean corpuscular hemoglobin (Table 1). The platelet count was low (52 × 10^9^/L), but the reticulocyte and the white blood cell (WBC) count were both within normal limits. Cobalamin was low, but not folate or ferritin. Apart from the hematological abnormalities, there was also low albumin as well as elevated levels of aspartate aminotransferase, lactate dehydrogenase, total bilirubin, conjugated bilirubin, high-sensitive troponin T, and pro-brain natriuretic peptide. Other blood tests were within normal values (Table 1). Further blood analyses revealed no monoclonal protein component in blood or urine. The patient was admitted to the hematological ward and transfused with two units of packed red blood cells. The next day, the RBC count had increased to 81 g/L and the general condition of the patient had improved slightly. She also received intravenous fluid (5% glucose) because of fasting for different examinations and vitamin B substitution (hydroxocobalamin, 1 mg daily as an intramuscular injection). She underwent a bone marrow aspiration and biopsy from the iliac crest that showed a hyperplastic marrow with greatly increased erythropoiesis but no genetic discrepancies suggestive of myelodysplastic syndrome or other hematological disease. No silver particles were identified during microscopic examination of the bone marrow. Computed tomography of the chest and abdomen revealed right-sided pleural effusion and small basal atelectases of the right lower lobe. The patient was subsequently drained of 550 mL of pleural fluid where laboratory analyzes of protein content, glucose, lactate dehydrogenase, RBC, and WBC supported that it was a transudative pleural effusion. Bacterial culture was negative, and cytology showed no malignant cells. A transthoracic echocardiography revealed a hyperdynamic left ventricle, borderline enlarged left atrium, and minor pericardial effusion without any hemodynamic significance. An electrocardiogram revealed normal sinus rhythm and no conduction abnormalities.

**Table 1:** Laboratory parameters from admission (day 0), inpatient care (day 4), and outpatient follow-up (day 44).

Laboratory parameter	Day 0	Day 4	Day 44	Normal values
**Hematology**				
B-red blood cell count (g/L)	48	81	129	117–153
B-mean corpuscular volume (fL)	153	111	95	82–98
B-mean corpuscular hemoglobin (pg)	51	37	30	27–33
B-reticulocytes (10^9^/L)	106	143	65	36–112
B-white blood cell count (10^9^/L)	5.6	5.5	7.2	3.5–8.8
B-basophils (10^9^/L)	0.01	0.05	0.06	< 0.1
B-eosinophils (10^9^/L)	0.1	0.2	0.2	< 0.5
B-neutrophils (10^9^/L)	3.6	3.6	4.8	1.6–5.9
B-monocytes (10^9^/L)	0.28	0.5	0.5	0.2–0.8
B-lymphocytes (10^9^/L)	1.5	1.1	1.7	1.1–3.5
B-platelet count (10^9^/L)	52	50	249	165–387
P-cobalamin (pmol/L)	< 75	> 1475	858	145–569
P-folate (nmol/L)	13	5	45	7–39
P-ferritin (µg/L)	159	162	140	30–400
**Hemostasis**				
P-activated partial thromboplastin time (s)	23.3	25.2	23.4	20.0–29.0
P-prothrombin time (INR)	1.2	1.1	1.1	< 1.3
**Fluid and electrolyte balance**				
P-sodium (mmol/L)	143	149	142	137–145
P-potassium (mmol/L)	4.0	3.4	3.7	3.5–4.4
P-corrected calcium (mmol/L)	2.2	2.2	2.4	2.2–2.5
P-albumin (g/L)	30	25	35	36–45
P-creatinine (µmol/L)	59	45	57	45–90
**Liver function**				
P-aspartate aminotransferase (ukat/L)	1.0	0.4	0.4	< 0.6
P-alanine aminotransferase (ukat/L)	0.2	0.3	0.2	< 0.8
P-gamma glutamyl transferase (ukat/L)	0.2	0.2	0.4	< 1.2
P-alkaline phosphatase (ukat/L)	0.8	0.7	1.1	0.7–1.9
P-lactate dehydrogenase (ukat/L)	34	19.3	3.5	< 3.4
P-total bilirubin (µmol/L)	87	21	8	< 25
P-conjugated bilirubin (µmol/L)	21	10	< 2	< 5
**Heart function**				
P-Troponin T (ng/L)	26	-	-	< 14.5
P-proBNP (ng/L)	996	646	302	< 150

P: plasma, B: whole blood.

During a more thorough interview in the ward, the patient informed the staff that she had been drinking colloidal silver as a remedy for upper respiratory symptoms that she had experienced the preceding weeks. The product was marketed as a water disinfectant (Chemical Abstracts Service Number 7440-22-4) and was reported to consist of deionized water and a suspension of 99.99% pure silver particles in a concentration of 10 µg/mL. She had ingested one tablespoon (15 mL) of the product daily for two to three weeks. Before that, she had used the product more sporadically over several years. Following this information, blood and urine samples were collected and analyzed for metals. The samples were sent to an external laboratory that performed analyzes on whole blood and a spot urine sample using inductively coupled plasma sector field mass spectrometry (ICP-SFMS) according to appropriate standards (ISO 17294-2:2016 and US EPA 200.8:1994). The samples were prepared using nitric acid and microwave heating according to SE-SOP-0412. The silver concentration in whole blood was 20.0 µg/L (measurement uncertainty ± 2.0 µg/L) and in urine 10.0 µg/L (± 1.0 µg/L). The patient was strongly advised to discontinue the intake of colloidal silver and was discharged from the hospital after ten days. During outpatient follow-up, she continued to improve and felt completely recovered after about six weeks, at which point cobalamin supplementation was discontinued. At that time (day 44), most blood parameters had normalized, and the RBC count was 129 g/L (Table 1). The pro-brain natriuretic peptide had decreased (302 ng/L), and there were no clinical signs of decompensation. The silver analysis was repeated after 95 days from the baseline measurement and the whole-blood concentration had decreased to 3.3 µg/L, corresponding to a calculated half-life of 37 days.

## 3 Discussion

Oral silver poisoning in humans has seldom been reported in the literature. Inadvertent ingestion often entails low doses dispersed over long periods, such as when eating contaminated seafood and fish. Voluntary ingestion has mainly been described in more recent years with the advent of colloidal silver suspensions as alternative medicine treatments for different ailments [2], [3], [4].

Our patient presented with lethargy, early signs of heart failure, abnormal liver function tests as well as severe anemia and thrombocytopenia. According to the literature, adverse effects of silver are mainly seen in the cardiovascular, hepatic, and hematopoietic systems [1], [2]. In animal studies, silver exposure has induced cardiac enlargement and ventricular hypertrophy [5] as well as hepatic complications (e.g., centrilobular necrosis) [6], [7]. Pleural effusion has also been described [8]. Anemia of the microcytic hyperchromic type has been described in animals chronically exposed to dietary silver compounds [5], [9], [10], while very little is known about the effects on the human hematopoietic system [1]. In one case report regarding colloidal silver ingestion, normocytic anemia was found, but no further details were presented [11]. In our patient, the RBC indices instead indicated a macrocytic, hyperchromic type of anemia. Silver is thought to have a direct toxic effect on the bone marrow [12], and it is possible that the MCV could be both increased and decreased as a sign of abnormal erythropoiesis. In animal studies, the administration of silver nanoparticles has been shown to induce reactive oxygen species and DNA damage [13], with effects most prominently seen in bone marrow polychromatic erythrocytes [14]. In an in vitro study on human mesenchymal stem cells exposed to silver nanoparticles, uptake and localization of such particles was seen in the cytoplasm. Following the internalization of silver nanoparticles, there was an increase in reactive oxygen species, signs of DNA damage, reduced mitochondrial membrane potential, and reduced cell viability as a result of both apoptosis and necrosis [15]. In our patient, there were also signs of hemolysis, with increased lactate dehydrogenase and bilirubin. Other heavy metal intoxications such as lead and arsenic have been reported to induce hemolysis, but little is known about the hemolytic potential of silver [1], [8]. Our patient also had thrombocytopenia, and this is a finding that goes well with a general toxic effect of silver on the bone marrow, where even agranulocytosis has been described [8]. Moreover, our patient showed signs of cobalamin deficiency but reported no abnormal diet and a thorough medical investigation revealed no indications of malabsorption. This vitamin deficiency could explain the macrocytic tendency and be a parallel condition to the silver intoxication, but it is also possible that it reflects an interaction with the orally ingested silver compound. Previous reports on silver have documented interactions with the uptake of selenium, copper, and vitamin E, but there is yet insufficient knowledge on the effects of cobalamin uptake [1]. Our patient was primarily treated symptomatically with blood transfusion and hydroxocobalamin injections and improved rapidly over about ten days. In the literature, no effective specific therapy for silver poisoning has been described [1] and chelation therapy has proved ineffective [16], [17]. Complete cessation of silver ingestion has been described as paramount [4].

Regarding exposure, our patient had ingested about 150 µg of colloidal silver daily. This is much higher than the 1–16 ug that has been suggested as a normal oral daily intake in Sweden [18]. Silver compounds have been reported to be absorbed by 10–20% after ingestion, though most data once again come from animal studies [1]. After ingestion, silver compounds are mostly deposited in the liver and spleen, but can also be found in skeletal muscles, skin, and brain. In body fluids, silver binds to high molecular weight proteins as well as metallothionein, and is excreted mainly through biliary pathways [1]. Assessment of silver concentration in whole blood has been described as the most reliable method for determining recent exposure [1]. The whole-blood concentration of 20 µg/L in our patient was high when compared to the few previous publications on the topic. In a study of occupationally exposed silver factory workers (N = 30), the mean concentration in whole blood was 11 µg/L (interval 6–26 µg/L) while an unexposed control group (N = 35) had mean levels of less than 5 µg/L [19]. In another study performed in the occupational setting, silver reclamation workers (N = 19) had a mean concentration of 6.8 µg/L (interval 1.3–20.0 µg/L), silver factory workers (N = 70) 2.5 µg/L (interval 0.1–16 µg/L), jewelry production workers (N = 9) 1.2 µg/L (interval 0.2–2.8 µg/L), and unexposed farmers less than 0.1 µg/L [20]. Most of the silver exposure in these occupational cohorts was airborne, although the mucociliary propulsion of particles from the upper airways to the pharynx can result in some silver particles also being orally ingested [20]. In more recent case reports of patients with clinical argyria after voluntary ingestion of colloidal silver, the blood concentration has ranged from 11–29 µg/L [3], [4]. In our patient, the whole-blood silver concentration decreased over the follow-up of about three months, and the calculated half-life was 37 days. This can be compared to a biological half-life in human subjects of about 50 days described in the literature [21], [22]. The kinetics vary substantially between tissues, and deposits in the skin are likely to be the most long-standing [1]. Human and animal data suggest that 1–5% of silver is more permanently retained in the tissues [19]. Our patient showed no clinical signs of deposition of silver in the skin, mucus membranes, or cornea, such as in argyria. This might be due to the fact that she had ingested relatively high concentrations of silver during a shorter period and thus not developed tissue deposition, which has been described as a rather chronic effect [20]. Moreover, our patient also had a silver concentration of 10 µg/L in a spot urine sample. There is very limited data in the literature for comparison, but one study reported a range of 6–15 µg of daily urinary excretion among exposed individuals [23], while unexposed individuals generally had less than 2 µg of daily excretion [24]. In a recent case report of argyria, the urine concentration was 12.8 µg/L [3], closely resembling the finding in our patient. It has been suggested that urinary analysis is only relevant if the exposure has recently been very high [20], [24], and that fecal concentration is a better alternative for determining the total body burden of silver [19].

## 4 Conclusions

The case demonstrates the potential effects of silver intake on the hematopoietic, hepatic, and cardiovascular systems. This highlights the possible risks associated with emerging alternative medicine therapies.

## Funding

This research did not receive any specific grant from funding agencies in the public, commercial, or not-for-profit sectors.

## Declaration of Competing Interest

The author declares that he has no known competing financial interests or personal relationships that could have appeared to influence the work reported in this paper.

## Data Availability

The authors do not have permission to share data.
